# Employment status and health related quality of life among Hodgkin-lymphoma survivors’– results based on data from a major treatment center in Hungary

**DOI:** 10.1186/s12955-017-0758-x

**Published:** 2017-09-19

**Authors:** Ferenc Magyari, Karolina Kósa, Roland Berecz, Anna Illés, Zsófia Miltényi, Zsófia Simon, Árpád Illés

**Affiliations:** 1 Department of Hematology, Faculty of Medicine, University of Debrecen, Debrecen, Hungary; 2 Department of Behavioural Sciences, Faculty of Public Health, University of Debrecen, Debrecen, Hungary; 3 Department of Psychiatry, Faculty of Medicine, University of Debrecen, Debrecen, Hungary; 4 Department of Anaesthesiology and Intensive Care Faculty of Medicine, University of Debrecen, Debrecen, Hungary

**Keywords:** Hodgkin-lymphoma, Employment, HRQoL, Survivorship, Long-term side effects

## Abstract

**Background:**

Due to risk and response adapted treatment strategies, more than 80% of newly diagnosed classical Hodgkin lymphoma (HL) patients can be cured, and become long-term survivors. However, a high proportion of survivors suffer from treatment-related long-term side effects such as secondary malignancy, organ failure, persistent fatigue and psychological distress. The aim of this study was to evaluate psychological distress and its risk factors among our HL survivors.

**Methods:**

One hundred sixty-three (50% female) adult HL survivors were contacted between January 1, 2012 and march 31, 2015 in our outpatient centre. The patients were asked to complete a standardized, validated, self-administered Hungarian questionnaire with demographic questions and the following scales: Hospital anxiety and depression scale (HADS14), general health questionnaire (GHQ12), sense of coherence (SOC13) perceived stress scale (PSS4), dysfunctional attitude scale (DAS17). Disease and treatment data were acquired from hospital records.

**Results:**

Majority of HL survivors are in early adulthood, our most important goal should be to return them to normal life after their lymphoma is cured. The employment status at the time of survey seemed to be crucial so patients were divided into either active (*n* = 93) or inactive (*n* = 47) group. Retired survivors (*n* = 19) were excluded from the subgroup analysis. Psychological distress was significantly lower in active patients. Multiple logistic regression analysis showed significant differences between the inactive and active subgroups, such as age at diagnosis (≥30 years or below, *p* = 0.001), education level (below college vs. college, *p* = 0.032) and treatment related long-term side effects (yes vs. no, *p* < 0.001). Predictors for treatment-related long-term side effects are female gender (*p* = 0.011), chemotherapy protocol (ABVD vs. other, *p* < 0.001).

**Conclusions:**

Our data suggest that employment status and treatment-related long-term side effects play a critical role in the health related quality of life outcome among Hungarian HL survivors.

## Background

Due to risk and response adapted treatment strategies, more than 80% of newly diagnosed Hodgkin lymphoma (HL) patients at any stage can be cured, and are expected to be long-term survivors [1–3]. The majority of HL patients are in early adulthood (median age about 30 years), so the quality of life after treatment closure is of fundamental importance. Lymphoma survivors are live longer, and consequently are at risk of experiencing adverse psychical and psychosocial side effects for longer periods of time due to their disease and its treatment [4]. Treatment related long-term side effects such as secondary malignancy, or organ failure reduces the quality of life and life expectancy of survivors. There is a growing number of promising studies focused on survival related issues in former HL patients, mainly addressing overall health-related quality of life (HRQoL) [5]. HRQoL as a multidimensional construct is based on the World Health Organization’s definition of health incorporating physical, mental, and social aspects of health [6]. HRQoL may be impaired due to treatment-induced organic dysfunctions, psychological consequences, fatigue, persisting gonadal and cognitive dysfunction, difficulties in social and role functioning in HL survivors. HRQoL is determined based on patient reported outcomes [7]. The purpose of our study was to evaluate the frequency of psychological distress and its risk factors among HL survivors based on our single centre experience.

## Methods

### Subjects and data collection

A cross-sectional survey was conducted at the Department of Hematology of the University of Debrecen. We designed the study for approximately 150 from the 301 regularly followed up patients. Exclusion criteria were age under 18 years at the time of survey and a lack of signed informed consent. 170 patients were asked to participate in our study. The refusal rate was below 5%. Altogether, 163 adult HL survivors were identified between January 1, 2012 and March 31, 2015 in our outpatient clinic whose diagnosis of HL was established between January 1, 1969 and July 1, 2013. Diseases diagnosed before the initiation of HL treatment were defined as comorbidities, whereas treatment related side effects were defined as conditions diagnosed in the follow-up phase after HL treatment based on hospital records of the patients.

### Treatment protocols

Primary chemotherapy basically involved CV(O)PP (cyclophosphamide, vinblastine [vincristine], procarbazine and prednisolone) before. After 1990, COPP/ABV (cyclophosphamide, vincristine, procarbazine, prednisolone/adriamycin, bleomycin and vinblastine) was used. Since 1999, the ABVD (adriamycin, bleomycin, vinblastine and dacarbazine) protocol has been most frequently applied. In cases of relapse, treatment involved BEACOPP (bleomycin, etoposid, adriamycin, cyclophosphamide, vincristin, procarbazine and prednisolone), DHAP (dexamethasone, cytarabine and cisplatin), or CEP (CCNU, etoposide, chlorambucil and prednisolone) regimens. Radiotherapy of the involved or extended field, mantle, inversed Y or (sub)total nodal type was administered by a telecobalt device before 2000 and more recently by a linear accelerator. Extended and involved field radiotherapy were used before (mean dose, 40 Gy) and after 1998 (mean dose, 33 Gy), respectively (Table 1).

**Table 1:** The treatment protocols based on the stage of disease

Treatment	Number of patients				
	Stage I.	Stage II.	Stage III.	Stage IV.	All
ABVD/EBVD + RT	3	52	9	8	72 (51.5%)
ABVD/EBVD – RT	0	5	9	9	23 (16.5%)
COPP/ABVD + RT	1	3	12	0	16 (11%)
COPP/ABVD – RT	0	0	5	2	7 (5%)
CVPP + RT	1	6	4	1	12 (9%)
CVPP – RT	1	0	2	0	3 (2%)
Other	1	2	4	0	7 (5%)

A(E)BVD: adriamycin (epirubicin), bleomycin, vinblastine and dacarbazine; CV(O)PP: cyclophosphamide, vinblastine [vincristine], procarbazine and prednisolone; COPP/ABV: cyclophosphamide, vincristine, procarbazine, prednisolone/adriamycin, bleomycin and vinblastine; Other protocols include MOPP: mustargen, vincristine, procarbazin, prednisolone, OEPA: vincristine, etoposide, prednisone, and doxorubicin

### Questionnaires

HL survivors were asked to complete a standardized, self-administered and validated Hungarian questionnaire, which included items on socio-demographic status (place of residence, marital status, educational level, employment, important life events after lymphoma treatment) and psychiatric treatment (date and type of medication) at the time of diagnosis, as well as the scales listed below. Data on the disease and its treatment were based on hospital records.

The Hungarian version of the **Hospital Anxiety and Depression scale** (HADS-14) has been used in studies of distress among cancer patients in general. Each of the 14 items is scored on a 4-point scale (0–3). Sum scores for the anxiety and depression subscales are calculated by simple addition. The constructors of HADS recommended two possible cut-offs (8 or higher or 11 or higher on either scale) for case definition. In this study, caseness refers to the lower cut-off [8, 9]. Due to printing error item 10 in the HADS questionnaire was missing answer 2 and 3.

**The General Health Questionnaire** (GHQ-12) is the most extensively used screening instrument for common mental disorders, in addition to being a general measure of psychiatric well-being. Each item is scored on a 4-point scale (corresponding to a symptom present: ‘not at all’, ‘same as usual’, ‘rather more than usual, or ‘much more than usual’). It can be scored in a bimodal fashion (0–0–1-1), when final scores range from 0 to 12. According to this method, patients scoring 5 or more are considered: at risk of anxiety/depression [10].

The validated Hungarian version of the abbreviated **sense of coherence** (SOC-13) scale was used in the present survey to measure the overall capacity to cope with stressful situations. All 13 items are answerable on a Likert scale from 1 to 7, total scores vary between 13 and 91. A higher score indicates stronger SOC [10].

**The Perceived Stress Scale** (PSS) is the most widely used psychological instrument for measuring perception of stress. Scores for the 4-item form range from 0 to 16. Potential responses range from 0 (never) to 4 (very often) and positively stated items are reverse coded before items are summed up with higher scores indicating more perceived stress [11].

**The Dysfunctional Attitude Scale form A** (DAS-A) is designed to measure the presence and intensity of dysfunctional attitudes. The higher the score, the more dysfunctional attitudes are characteristic of an individual. The 17 items are divided into two subscales: perfectionism and dependency. Each item is scored on a Likert scale from 1 to 7. Sum scores for either subscale are calculated by simple addition [12].

### Statistical analysis

Statistical analysis was performed using IBM SPSS 20 software. Data are described by the mean, standard deviation frequencies and percentages. Categorical variables were compared between groups using chi-squared or Fisher’s exact test, as appropriate. Continuous variables were evaluated by independent samples t-test, Mann-Whitney test and ANOVA or Kruskal-Wallis test. Spearman’s correlations were used measuring the relationship between two variables. Significance level was set at *p* < 0.05. Multiple logistic regression (backward Likelihood Ratio method) was performed among HL survivors to identify predictors of employment status Odds ratios (OR) with 95% confidence intervals were estimated for the logistic regression models [13].

## Results

### Patient characteristics

A total of 163 adult HL survivors completed the survey. 19 persons were beyond retirement age of 65 years and 4 persons had unknown employment status who therefore were excluded from the subgroup analysis. Data of 140 HL survivors (71 females, 51%) of working age were investigated and the characteristics of survivors are presented in Tables 1 and 2. The mean ages of survivors at the time of diagnosis were 32.13 ± 13.05 years and at completion of the survey 44.82 ± 14.55 years. 57 survivors (41%) had mixed cell HL, which was the most frequent histological subtype. 73 patients (53%) had stage I-II and 65 patients (47%) stage III-IV HL. 68 survivors (49%) had baseline comorbidity (most common: cardiovascular disease 11%, gastroenterological 10%, dermatological 5%, musculoskeletal 5% and haematological 2% of patients). 95 patients (69%) had received ABVD treatment, while 43 patients (31%) had received some other type of chemotherapy. Radiation had been administered to 103 patients (74%).

**Table 2:** Characteristics of survivors of working age and independent variables of employment by the results of multiple logistic regression analysis

	All patients (*n* = 140)	Inactive employment status (n = 47)	Active employment status (n = 93)	Univariate analysis			Multiple analysis		
				*p*	ODDS	95% CI	*p*	ODDS	95% CI
Age at survey > = 40 yr.	76 (54%)	15 (32%)	61 (66%)	<0.001	4.357	2.038–9.316			
vs. <40 yr.	64 (46%)	32 (68%)	32 (34%)						
Age at dg. > = 30 yr.	80 (57%)	**16 (34%)**	**64 (69%)**	**<0.001**	**4.276**	**2.028–9.016**	**0.001**	**3.556**	**1.548–8.168**
vs. <30 yr.	60 (43%)	**31 (66%)**	**29 (31%)**						
Elapsed time > =10 yr.	66 (47%)	24 (51%)	42 (45%)	0.436	1.325	0.652–2.689			
vs. <10 yr.	74 (53%)	23 (49%)	51 (55%)						
Male	69 (49%)	23 (49%)	46 (49%)	0.953	0.979	0.485–1.975			
vs. female	71 (51%)	24 (51%)	47 (51%)						
Residence: village	32 (23%)	12 (26%)	20 (22%)	0.592	1.252	0.507–2.062			
vs. city	108 (77%)	35 (74%)	73 (78%)						
Education level:									
elementary	50 (36%)	**24 (53%)**	**26 (28%)**	**0.014**	**3.346**	**1.282–8.734**	**0.032**	**3.320**	**1.112–9.914**
vs. college	37 (27%)	**14 (30%)**	**38 (41%)**						
elementary				0.568	1.336	0.494–3.609	0.235	1.967	0.644–6.007
vs. high school	52 (37%)	8 (17%)	29 (31%)						
Single	68 (50%)	23 (52%)	45 (49%)	0.714	1.144	0.557–2.347			
vs. live together	68 (50%)	21 (48%)	47 (51%)						
Stage 3–4	73 (53%)	24 (52%)	41 (45%)	0.399	1.357	0.668–2762			
vs. 1–2	65 (47%)	22 (48%)	51 (55%)						
Bulk	42 (32%)	11 (27%)	31 (34%)	0.409	0.710	0.314–1.605			
vs. without bulk	90 (68%)	30 (73%)	60 (66%)						
B symptom	61 (46%)	20 (49%)	41 (44%)	0.691	1.161	0.555–2.433			
vs. no B symptom	71 (54%)	21 (51%)	50 (56%)						
ECOG 1–2	22 (17%)	8 (20%)	14 (16%)	0.593	1.299	0.497–3.390			
vs. ECOG 0	108 (83%)	33 (80%)	75 (84%)						
Comorbidity	68 (49%)	25 (53%)	43 (44%)	0.437	1.321	0.654–2.667			
vs. no comorbidity	72 (51%)	22 (47%)	50 (56%)						
Treatment: ABVD	43 (31%)	16 (34%)	27 (30%)	0.599	1.224	0.576–2.597			
vs. other	95 (69%)	31 (66%)	64 (70%)						
Irradiation	103 (74%)	35 (74%)	68 (73%)	0.864	1.072	0.482–2.387			
vs. no irradiation	37 (26%)	12 (26%)	25 (27%)						
Treatment response: PR	72 (51%)	4 (9%)	5 (5%)	0.484	1.637	0.418–6.409			
vs. SD	68 (49%)	43 (81%)	88 (95%)						
Treatment related side effect: yes	72 (51%)	**36 (77%)**	**36 (39%)**	**<0.001**	**5.182**	**2.343–11.460**	**<0.001**	**5.254**	**2.196–12.524**
vs. no	68 (49%)	**11 (23%)**	**57 (61%)**						
Relapse	21 (15%)	7 (15%)	14 (15%)	0.980	0.983	0.369–2.641			
vs. no relapse	119 (85%)	40 (85%)	79 (85%)						

The most common treatment related long-term side effects were examined in active vs. inactive groups: cardiovascular disease 9 (10%) vs. 12 (25%) patients (*p* = 0.026), pulmonary disease 7 (8%) vs. 6 (12%) patients, thyroid disease 23 (25%) vs. 17 (36%) patients and post-irradiation skin disorder 4 (4%) vs. 4 (8%) patients, respectively.

### Employment status

**Fig. 1:**
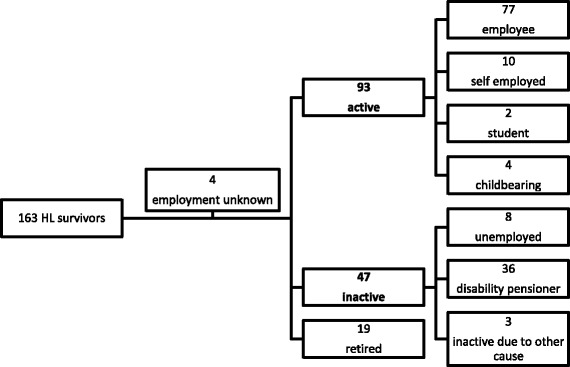
Survivors’ employment status at completion of survey (organogram)

**Fig. 2:**
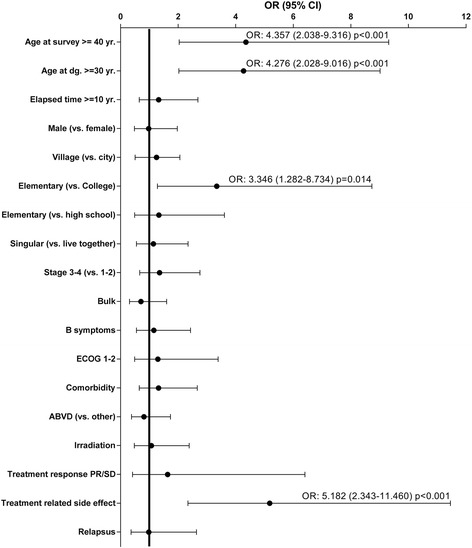
ORs and 95% CI of inactive vs. active employment status by clinical data in HL survivors of working age. The analysis was performed by binary logistic regression. Values under 1 are related to the active employment status, above 1 to inactive employment status

The majority of HL survivors had been in early adulthood at the time of diagnosis. Hence, the most important goal for them is to return to normal life including work after their lymphoma is cured. Employment at the time of the survey was defined as crucial factor of normal life so the survivors were split into two subgroups based on employment status: active (93 patients) and inactive (47 patients, see Fig. 1). Age at diagnosis of HL (*p* < 0.001), completion of the survey (p < 0.001), educational level (*p* = 0.017) and treatment-related long-term side effects (*p* < 0.001) were significantly different between the inactive and active subgroups as shown in Table 2. Employed survivors were on average 10 years younger at the time of the survey, they had their diagnosis 7.5 years earlier, almost twice as many of the had college degrees, and half of them had treatment related long-term side effects compared to the inactive group. Odds ratios and 95% CI of inactive versus active employment status by HL survivors’ clinical data are presented in Fig. 2.

### Mental health of survivors

Thirty-four survivors (25%) had caseness scores with HADS anxiety and 14 (10%) with HADS depression. However, means for HADS anxiety and depression, perceived stress (PSS), stress-depression-anxiety (DAS total) and perfection as a dysfunctional attitude (DAS perfection) were found to be significantly higher among inactive HL survivors. 14 (10%) of HL survivors had abnormal levels of distress with GHQ, but 5 times more of them were inactive compared to the active ones. In terms of sense of coherence as a measure of generalised coping resources, active survivors had significantly higher mean score. With the exception of dependency, as a dysfunctional attitude, all other measures reflected considerably less favourable mental health among those who had been inactive. The results of mental health questionnaires are shown in Table 3.

**Table 3:** Quality of Life questionnaires for survivors

	All patients (*n* = 140)	Inactive(I) (*n* = 47)	Active(A) (*n* = 93)	P (I-A)
	Mean ± SD			
HADS anxiety	5.46 ± 3.54	**6.69 ± 3.64**	**4.87 ± 3.35**	**0.004**
HADS depression	3.05 ± 3.19	**4.87 ± 3.84**	**2.17 ± 2.38**	**<0.001**
PSS	4.55 ± 2.80	**5.93 ± 2.83**	**3.87 ± 2.53**	**<0.001**
SOC	66.90 ± 11.26	**62.55 ± 69.03**	**69.03 ± 10.51**	**0.002**
DAS total	49.35 ± 18.94	**54.79 ± 22.01**	**46.80 ± 16.86**	**0.022**
DAS perfection	28.41 ± 12.61	**31.98 ± 14.94**	**26.74 ± 11.07**	**0.024**
DAS dependency	20.94 ± 8.01	22.81 ± 8.35	20.07 ± 7.74	0.063
	Number of patients (%)			
HADS anxiety				
normal score: 0–7	**104 (75%)**	**26 (57%)**	**78 (84%)**	**0.004**
borderline: 8–11	**21 (15%)**	**12 (27%)**	**9 (10%)**	
abnormal: 11–21	**13 (10%)**	**7 (16%)**	**6 (6%)**	
HADS depression				
normal score: 0–7	**124 (90%)**	**35 (78%)**	**89 (96%)**	**0.005**
borderline: 8–10	**8 (6%)**	**6 (13%)**	**2 (2%)**	
abnormal: 11–21	**6 (4%)**	**4 (9%)**	**2 (2%)**	
GHQ				
normal score: 0–4	**124 (90%)**	**35 (78%)**	**89 (96%)**	**0.002**
abnormal: ≥5	**14 (10%)**	**10 (22%)**	**4 (4%)**	

*HADS* Hospital Anxiety and Depression scale, *GHQ* General Health Questionnaire, *PSS* Perceived Stress, Scale, *SOC* Sense of Coherence Scale, *DAS* Dysfunctional Attitude Scale

We defined severe mental vulnerability as having abnormal scores on at least 3 tests according to which 15 survivors (7 inactive, 5 active, 3 pensioners) were identified as in urgent need of care. 2/3 of them (10 survivors) were referred to specialist (clinical psychologist/psychiatrist). 5 persons referred to the team psychologist. Subsequently, 3 of the 7 inactive patients returned to work.

### Independent predictive factors for employment status

Multiple logistic regression analysis was carried out by starting with all potential determinant variables and eliminating the non-significant ones. Significant differences between inactive and active groups were found regarding (being at least or over ≥30 year vs. below) at diagnosis (*p* = 0.001), education level (elementary school or above vs. college, *p* = 0.032) and treatment related long-term side effects (yes vs. no, *p* < 0.001; Table 2).

### Predictive factors for treatment related long-term side effects

Predictive factors for treatment related long-term side effects are presented in Table 4. Females had a higher rate of treatment-related long-term side effects compared to males (*p* = 0.011), those on ABVD regimen suffered more side effects compared to those who had received other regimens (*p* < 0.001). Relapse rate (*p* = 0.003) and baseline comorbidities (*p* < 0.001) were significantly higher among female survivors (71 patients) (not shown). Independent variables of treatment-related long-term side effects are female gender (OR: 2.67 (95% CI 1.327–5.375); *p* = 0.006) and other regimens than ABVD (OR: 6.17 (95% CI 2.788–13.655); *p* < 0.001).

**Table 4:** Predictors for treatment related long-term side effects

	Treatment related long- term side effects	No treatment related long-term side effects	*P*
Gender			
Male (*n* = 69)	28 (41%)	41 (59%)	0.011
Female (*n* = 71)	44 (62%)	27 (38%)	
Chemotherapy			
A(E)BVD (*n* = 95)	38 (40%)	57 (60%)	<0.001
Other than ABVD (*n* = 43)	33 (77%)	10 (23%)	

A(E)BVD: adriamycin, (epirubicin), bleomycin, vinblastine and dacarbazine; Other than ABVD: CV(O)PP:cyclophosphamide, vinblastine [vincristine], procarbazine and prednisolone; COPP/ABV:cyclophosphamide, vincristine, procarbazine, prednisolone/adriamycin, bleomycin and vinblastine

### Loss of work after HL treatment

**Fig. 3:**
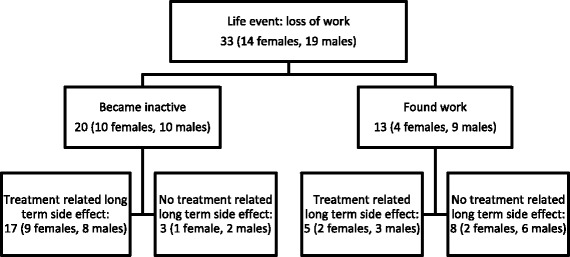
Loss of work after lymphoma treatment by patient reports

There were 13 survivors in the active group, who lost their work after treatment but later found new jobs. 20 survivors became inactive due to job loss in the inactive group of whom 17 persons as opposed to only 5 of the 13 in the active group had treatment-related long-term side effects (*p* = 0.009). These results are presented in Fig. 3.

## Discussion

The cure of Hodgkin lymphoma is one of the success stories of cancer medicine in the twentieth century. This resulted in a continuously growing number of HL survivors at risk for long-term sequel and quality impairment in their lives. The majority of HL survivors have been in early adulthood so their most important goal is to return to normal life once their disease is cured [7]. Work is a critical aspect of normal life so our study investigated long-term HL survivors by their employment status, excluding those over retirement age (65 years).

Two-third of our survivors was active at the completion of the survey. They had been significantly younger at the time of HL diagnosis as well as at the completion of the survey; they had higher educational level, and less treatment-related long-term side effects compared to inactive survivors. Neither gender, nor characteristics of the disease at the time of diagnosis were found to be different between the active and inactive groups. HL survivors’ employment status varies from 71% to 85% [14–17]; our data are close to the lower end of this range. Hungarian HL survivors have other options regarding work after treatment. 25% of all participants became disability pensioners that provide a steady albeit low income, a definitive advantage compared to employment for some patients. Unemployment rate in the adult population of the country according to data of the Hungarian Central Statistical Office (available at: https://www.ksh.hu/) was 7.2% in the North-eastern region of Hungary (for 2012 to 2015, among 40–49 age group, both gender), almost 1.5 times higher than in our study population (5%). In contrast, the ratio of inactive persons was 1.5 times higher among HL survivors (21% vs. 14.5%). (Both groups are of working age, but unemployed persons search for work and registered as such, inactive persons do not search for work.)

In light of the fact that the proportion of disabled HL survivors varies from 9% in Sweden [17] to 19% in Denmark [18], more effort needs to be put into helping Hungarian patients find their way back to work. Employment status, the mental health of long-term survivors of cancer (lymphoma) should also be a focus for research. Our study used various tools to investigate the mental status of HL survivors; a strong correlation was observed between employment status and psychological well-being. Active (working) survivors were significantly less depressed, less anxious, less perfectionist than their inactive peers; they proved to be much more resilient as reflected by their sense of coherence; and correspondingly, only one-fifth of them were pathologically distressed compared to the inactive ones.

The majority of cured HL patients survive without psychological distress at case level with HADS questionnaire [19]. Previous studies of anxiety and depression in lymphoma patients using HADS observed prevalence of anxiety between 15 and 42%, and depression between 4 and 35% [20]. The prevalence of anxiety and depression were 25% and 10% using HADS in our study population, well within the range of other studies. So far there have been few published data with GHQ for haematological disorders. Broers et al. reported results for patients 3 years after bone marrow transplant among whom 13% showed mental stress by the GHQ, a percentage comparable to that in the general population [21]. Harila et al. [22] found no difference in GHQ scores between long-term ALL survivors and controls. Our experience revealed that 10% of HL survivors had abnormal levels of distress with GHQ, with a much lower proportion among working survivors. Our findings with the SOC questionnaire are similar to those of Wettergren et al. [23] who found a mean of SOC (66.8 ± 11.2) in the Swedish population that was almost identical to the mean of our patients. There are no literature data with DAS and PSS scales in haematological disorders.

Five previous studies with clinical data (four of them questionnaire-based) investigated employment specifically in HL survivors. Fobair et al. reported in 1986 that male gender, depression, age at or over 30 years, energy loss, and current disease status correlated with the number of hours worked. Neither stage nor treatment type predicted time to return work [14]. Abrahamsen published data in 1998on HL survivors’ employment status according to which 95% of 557 patients treated between1971–1991 had returned to work after 18 months. Female predominance was observed among unemployed survivors during the follow-up period (64% of women and 85% of men worked after 18 months of the start of treatment) [24]. Chen et al. reported that male gender and scarring of the head and neck were risk factors for having been denied a job due to medical history [15]. Glimelius et al. highlighted in 2015 that advanced stage HL survivors treated with full-dose chemotherapy were at risk of work loss which was not explained by relapsed disease, secondary malignancies or cardiovascular disease [17]. Behringer et al. published favourable data on social reintegration and treatment outcome among HL survivors. Their analysis revealed a significant negative association of severe fatigue (sFA) and employment status in survivors: 5 years after therapy, 51% and 63% of female and male survivors, respectively, with sFA had been in employment or in professional education, compared with 78% and 90% without sFA, respectively (*p* < 0.001) [25].

According to our data, lower age at diagnosis, college-level education, and no treatment-related long-term side effects are independent predictive factors for employment after treatment comes to an end. The risks factors of treatment-related long-term side effects are female gender, chemotherapy other than ABVD. The reduction of dose and field of radiation therapy and the application of ABVD regimen had become more tailored for HL treatment that might have led to the reduction of treatment-related long-term side effects which became apparent in our study population after 2002. Treatment-related long-term side effects had a clearly negative impact on inactive employment status. According to our results, the frequency of cardiovascular disease is significantly higher among inactive survivors.

## Conclusion

Our data suggest that employment status, education and treatment-related long-term side effects (hypothyreodism, cardiomyopathy, coronary sclerosis, valvulopathy, pulmonary fibrosis, postirradiation skin and muscle atrophy) play a critical role in the quality of life of HL survivors. In order to help HL patients become not only survivors but fully functioning, cured persons capable of working, the further development of target-specific therapeutic modalities (anti-CD30 targeting with brentuximab-vedotin, programmed death 1 (PD-1) blockers) is needed along with clinical studies that include follow-up measurements of quality of life.

A growing number of studies provide evidence for the necessity of work rehabilitation after the cure of cancer (among them Hodgkin-lymphoma). Denmark has a widespread tax-financed welfare system which provides an option for persons with permanently reduced work capacity to retire due to their disability and be financially compensated [18]. In Sweden, multidisciplinary interventions promote a healthy work environment/lifestyle involving physical, psychological, and vocational components [17]. Social reintegration program has also been available for oncological patients in Hungary. However, the Hungarian follow-up guidelines do not include recommendations for work rehabilitation program of HL survivors. Based on our investigation, we suggested an extended social reintegration program for Hungarian HL patients also. A clinical psychologist as member of our haematological team provides psychological support to patients in our department. There exists an active Lymphoma Civil Patient Forum that provides numerous opportunities to increase our patients’ psychological well-beeing. Control examinations create time for encounters between haematologist and patients during which psychological guidance facilitates HL survivors to step out of the patient role.
